# Corporeal Metritis, with Abscess of Broad Ligament

**Published:** 1869-12

**Authors:** F. B. Norcom

**Affiliations:** Chicago, Ill.


### ARTICLE VII. — *Corporeal Metritis, with Abscess of Broad Ligament.* By F. B. NORCOM, M.D., Chicago, Ill.

MRS. M——, æt. 29, scrofulous and nervous temperament, came to see me March 30, 1869, having been under a physician's care last two weeks, from some obscure uterine disorder. She was the mother of one living child, but had suffered several years back from two or three abortions — whether due to outside interference I could not discover — and seemed never to have recovered entirely from these shocks to her system. She has been always troubled by the "whites," more or less, even before marriage, and her pelvic organs appeared to exhibit defective organization, from the fact that her menstrual flow was often irregular and painful, and she was subject to frequent pains in the lumbar and hypogastric regions. Previous to this visit, one year ago, I had prescribed for a severe menorrhagic attack, without, however, being allowed an examination, and at that time suspected some womb disease.

She is now very pale and anxious looking, with slight hectic flush; tongue coated thick; pulse 110, small and irritable; bowels very constipated; anæmia, and irregular sleep. She states that, about two weeks back, was taken with excessive and painful uterine hæmorrhage, accompanied by severe pain in small of back, above pubis, and inner thigh, followed in a few days by fever, a dull, deep-seated, dragging sensation in hollow of sacrum, frequent micturition, great feeling of prostration, swollen abdomen, excessively tender over region of womb, etc. She had literally crawled into the office, where, upon stating these facts, an examination was immediately made, and detected cervical lips soft and ragged; os open, so that forefinger could be easily passed to first joint; great tenderness of the body upon bimanual manipulation, somewhat thickened besides. The adjoining tissues were so hard and sensitive that I was inclined to believe in pelvic cellulitis accompanying this uterine trouble, but later developments showed simply womb and ligament principally involved. The sound entered freely some three inches, creating much distress upon being moved in uterine cavity; while viewed through the speculum the os looked red and mottled, its edges somewhat torn and abraded, and considerable mucus with few drops of blood exuded.

The diagnosis: abortion *within* third month, by instrumental interference, and doing sufficient damage to create a marked degree of corporeal metritis. She was ordered to go home and get into bed, and the bowels to be opened by warm enemata, opium freely administered; emollient vaginal injections; hop poultices; unirritating liquid diet; and absolute rest in horizontal posture. The second day, complaining of a constantly increasing dull pain in left iliac region, I made a careful examination, and detected a tumor, size of a hazel-nut, a little to left of womb, movable, tender, and hard as a marble. There was now a new and dangerous complication introduced, and little expectation of cure, or even relief, until the abscess should mature, and open into some natural passage, or externally, the latter rather improbable. As the pain and other physical symptoms increased daily *pari passû* with enlargement of the tumor, and my patient seemed very ill and alarmed. I called in Dr. H. W. Jones, who confirmed the view I took, and indorsed the remedial measures employed. Two days later, the abscess had reached the size of an ordinary walnut, very plain upon palpation, tender, throbbing, very hard and movable, and accompanied by pyogenic fever, with total loss of appetite, unrefreshing sleep, and much nervous and irritative debility.

The friends, fearing a fatal issue, begged another consultation, and Prof. J. A. Allen saw the case, recognized the pathological condition, and upheld the expectant and palliative treatment.

The third day after this consultation, the abscess not yet breaking, she lost her confidence in my management, called in another physician, who assured her that there were three tumors, and that her former physicians should not have allowed her to get into such a dilemma. My successor, however, arrived just in time to witness the evacuation of the abscess through the large bowel, and a rapid subsidence of the painful symptoms. She now began to convalesce, and ten days afterwards, though not restored to her wonted health—for she still suffered from some uterine inflammation—she was about, attending her domestic duties, cursing all doctors, but he who had cured by the "laying on of hands," and consigning us to places too hot to be mentioned to ears polite. *Sic transit gloria medici.*

I will mention here, *en passant*, that she never acknowledged to any of us that she had had this abortion, and always denied her having been in a pregnant condition.

